# Ventriculo-arterial (un)coupling in septic shock: Impact of current and upcoming hemodynamic drugs

**DOI:** 10.3389/fcvm.2023.1172703

**Published:** 2023-05-30

**Authors:** Zoé Demailly, Emmanuel Besnier, Fabienne Tamion, Olivier Lesur

**Affiliations:** ^1^ Medical Intensive Care Unit, UNIROUEN, INSERM U1096, CHU Rouen, Normandie Université, Rouen, France; ^2^ Department of Anesthesiology and Critical Care, UNIROUEN, INSERM U1096, CHU Rouen, Normandie Université, Rouen, France; ^3^ Centre de Recherche Clinique du CHU Sherbrooke, Sherbrooke, QC, Canada; ^4^ Départements de Soins Intensifs et de Médecine et Service de Pneumologie, Faculté de Médecine et des Sciences de la Santé, Université de Sherbrooke, Sherbrooke, QC, Canada

**Keywords:** ventriculo-arterial coupling, sepsis, hemodynamic drugs, norepinephrine (NE), dobutamine, levosimendan, β-blockers, apelin

## Abstract

Sepsis is an archetype of distributive shock and combines different levels of alterations in preload, afterload, and often cardiac contractility. The use of hemodynamic drugs has evolved over the past few years, along with the invasive and non-invasive tools used to measure these components in real time. However, none of them is impeccable, which is why the mortality of septic shock remains too high. The concept of ventriculo-arterial coupling (VAC) allows for the integration of these three fundamental macroscopic hemodynamic components. In this mini review, we discuss the knowledge, tools, and limitations of VAC measurement, along with the evidence supporting ventriculo-arterial uncoupling in septic shock. Finally, the impact of recommended hemodynamic drugs and molecules on VAC is detailed.

## Introduction

The hemodynamic management of septic shock is an evolving challenge guided by recommendations updated regularly. According to the 2021 Surviving Sepsis Campaign (SSC) guidelines, with a target for a mean arterial pressure (MAP) of 65 mmHg or greater, norepinephrine, dobutamine, vasopressin, and possibly angiotensin II are the recommended medications after volume optimization (1). The majority of these drugs act on the two major components of hemodynamic homeostasis, i.e., the vascular network and the cardiac pump. However, emerging data (i.e., goal-directed therapies) support the use of additional or alternative targets to MAP and/or cardiac output (CO) with more adaptive and efficient resuscitation guidance (2).

Ventriculo-arterial coupling (VAC) is the net result of complex and constant interactions between the cardiac pump and its downstream vascular network (3). The objective of VAC is to consider the function of the heart with its afterload (the downstream arterial tree) as a whole and not separately and ideally to quantify the overall efficiency of the system. This allows us to go beyond the known limits of the simple measurement of the left ventricular ejection fraction (LVEF) or peripheral vascular resistance and to consider the adequacy of one in relation to the other. Thus, VAC provides a more physiological and integrative understanding of the cardiovascular function and may be a key element in the characterization and even the management of shock. For more than half a century, as for other hemodynamic measurement devices, tools for assessing VAC have evolved from invasive to potentially more affordable non-invasive approaches. Nevertheless, VAC remains difficult to apprehend in a practical routine. Therefore, to better understand this essential concept, this mini review aims to briefly address the principles of VAC and outline the alterations during septic shock and the impact of current and experimental circulatory drugs. Faced with the general concept of VAC and using a systematic search for the PubMed bibliographic database in VAC published works using the following keywords, “ventriculo-arterial coupling”; “uncoupling”; “vascular elastance”; “end-systolic elastance”; “hemodynamic drugs”; and “septic shock,” this narrative mini review focuses on the interaction between the left ventricle and the systemic arterial circulation during septic shock. No specific consideration for fluid resuscitation and right ventricle function is displayed.

## General principles of ventriculo-arterial coupling: a core determinant of cardiovascular performance

Although the heart and systemic vascular network have different intrinsic properties, both aim to provide adequate organ perfusion (through the generation of an outflow volume with a given level of outflow pressure). There is little sense in studying one without taking into consideration the condition of the other. Thus, the VAC concept was first proposed in 1984 as a method to evaluate the mechanical efficiency of the cardiovascular system and the interaction between cardiac performance and vascular function (4). For a complete review, see Antonini-Canterin et al. (5).

### Ventricular properties

Ventricular performance can be characterized by its end-systolic elastance (Ees), which reflects the ability of the ventricle to eject a stroke volume (SV) through the variation of the end-systolic pressure. In brief, it reflects the adaptability of the ventricle to the variation of afterload. From a physiological point of view, it is determined by the slope of the line passing through the point of the end of systole and by the theoretical point of zero volume (V0) on a pressure/volume loop and may be summarized by $Ees=\frac{Pes}{\left(Ves-V0\right)}$, where Pes and Ves are end-systolic pressure and volume, respectively. The accurate determination of Ees is difficult to assess, but its value can be estimated by the single-beat determination method developed by Chen et al. (6).

### Arterial properties

Properties of the vascular system are defined by vascular elastance (Ea), reflecting the resistance and compliance of the main arteries. Ea may be determined from the slope that joins end-diastolic volume and pressure and may be summarized as Ea = Pes/SV (7).

### VAC estimation

The Ea/Ees ratio is called the VAC index, reflecting the systolic performance of the left ventricle in front of the arterial compliance. Several publications estimated that the optimal VAC values are comprised of between 0.6 and 1.2 (3) or 0.5 and 1.3 (8). Beyond 1.3 implies ventriculo-arterial uncoupling and thus unbalance between reduced arterial compliance and ventricular inefficiency, which is appealing to therapeutic intervention (9).

**Figure 1:**
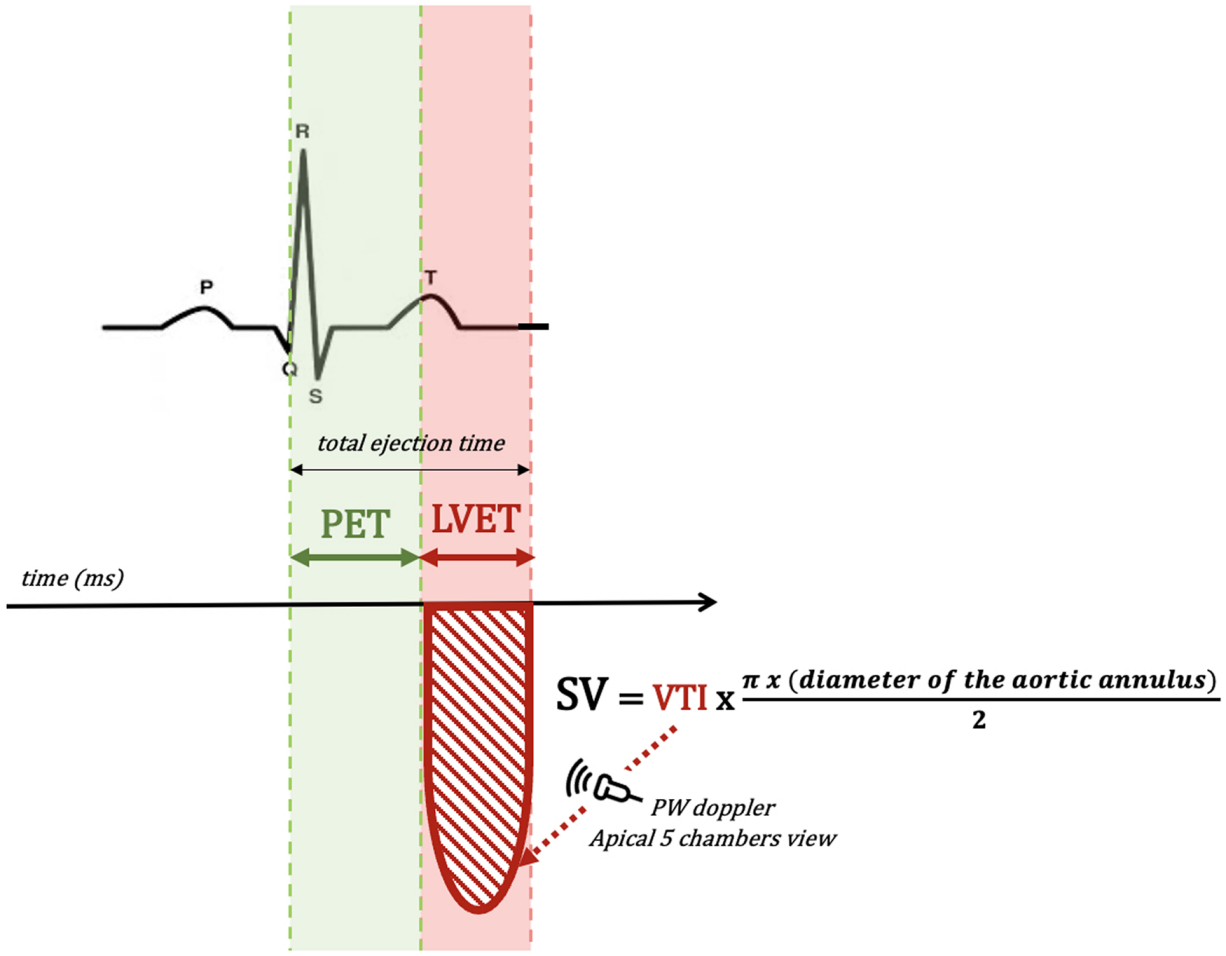
Schematic representation of echocardiographic measurements of PET, LVET, and SV. LVET, left ventricle ejection time; PET, pre-ejection time; SV, stroke volume; VTI, velocity–time interval.

Despite several proposed non-invasive approaches to evaluate VAC, only the modified single-beat method developed by Chen et al. (6) has been validated against the invasive measurement of *Ees*. This method utilizes the echocardiographic measures of LVEF, Doppler-derived SV [using the formula: SV = velocity–time interval (VTI, cm) × area (cm^2^), with VTI obtained using pulsed wave Doppler in the aortic wave during systole in a five-chamber view, and area = π × (diameter of the aortic annulus)/2, where the diameter of the aortic annulus is measured in the parasternal long-axis view during systole)], the estimated normalized ventricular elastance at the onset of ejection [End(est)], and the pre-ejection time (PET = time interval from the beginning of the QRS complex to the opening of the aortic valve) to left ventricle ejection time (LVET = time interval from the opening of the aortic valve to the closure of the aortic valve) ratio (tNd) (Figure 1), coupled with non-invasive systolic blood pressure (SBP) and diastolic blood pressure (DBP) measurements. The equations used are (6):

Ees=(DBP–[End(est)×SBP×0.9])/End(est)×SV

$Ea=\frac{\left(SBP\times 0.9\right)}{SV}$

with

$$\begin{array}{rl} End(est)= & \;\;0.0275\text{–}0.165\times LVEF+0,3656\\ & \times (DBP/(SBP\times 0,9))+0,515\times End(avg) \end{array}$$

$$End(avg)=0.35695-7.2266\times tNd+74.249\times tNd^{2}-307.39\times tNd^{3}+684.54\times tNd^{4}\text{–}856.92\times tNd^{5}+\;571.95\times tNd^{6}-159.1\times tNd^{7}$$

tNd=PET/LVET

The iElastance^©^ application is a simplified method to evaluate the VAC value by Chen et al.'s method without performing the calculations by hand (10).

## Sepsis-induced ventriculo-arterial uncoupling

**Figure 2:**
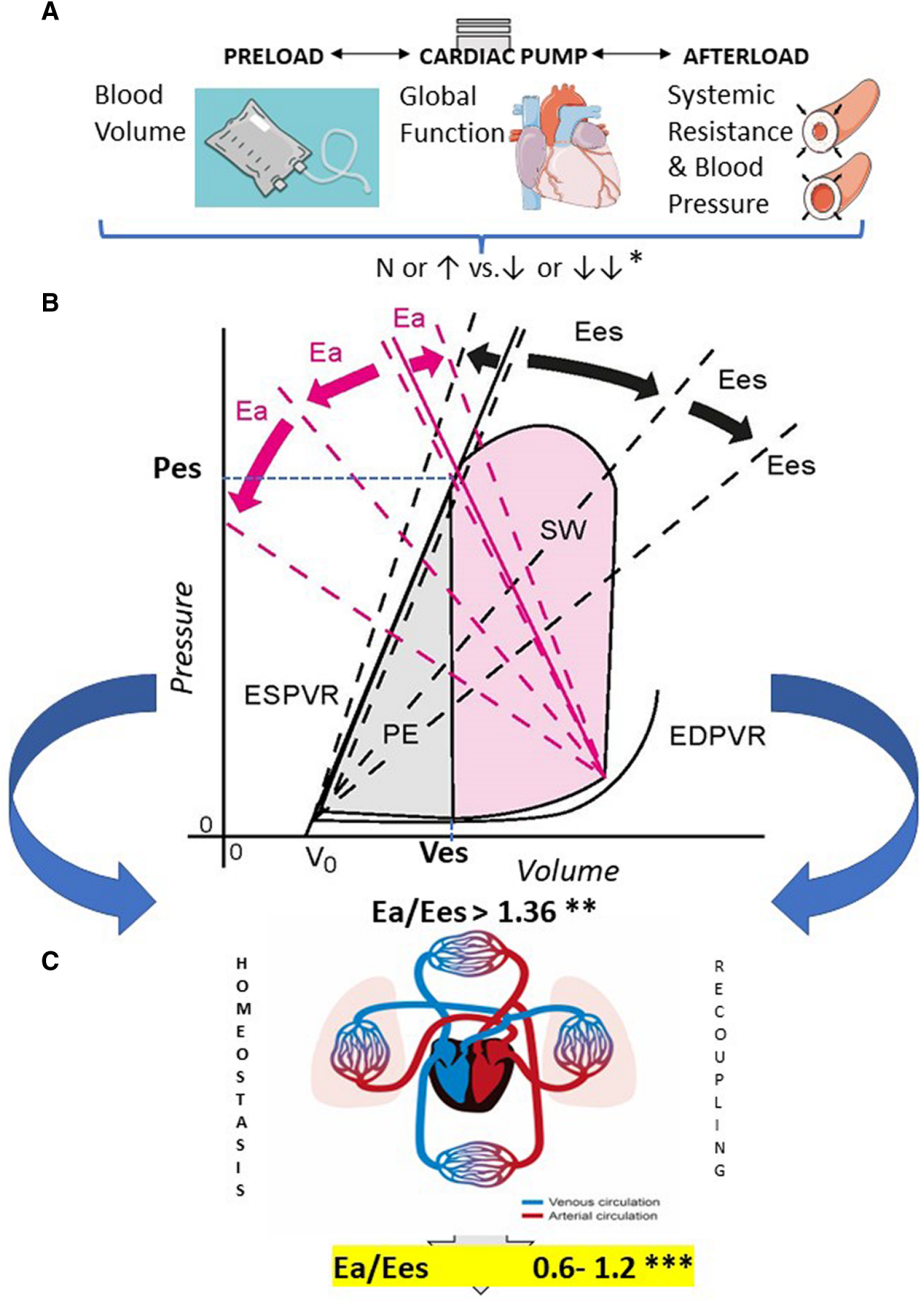
Early (acute) sepsis hemodynamics and ventriculo-arterial uncoupling on admission. From top to bottom: (**A**) The main components of sepsis-induced hemodynamic instability are first pictured out: *Relative percentages of patients: blood volume, 35%–45% *N* to↑ and 55%–65%↓ or ↓↓; global heart function, 75%–80% *N* to↑ and 20%–25% ↓ or ↓↓; systemic resistance and blood pressure, 10%–15% *N* to↑ and 85%–90% ↓ or ↓↓, as reported in ref. (13–21). (**B**) This comes down into counter-parted alterations in P/V loop parameters overall leading to ventriculo-arterial (VA) uncoupling which is characterized by variable unmatched changes of arterial elastance -Ea- and/or end-systolic left ventricular elastance—Ees-, moving up the ratio above 1.36, as defined by Guarracino et al. (22, 23). Prevalence of VA uncoupling has been observed in more than one- to more than three-quarters of early septic shock patients, depending on the study. **Relative percentages of patients with VA uncoupling: 25%–85%, as reported in ref. (12, 22–26). Potential moves of Ea (pink) and Ees (black) slopes are exemplified in dashed lines. (**C**) A priority with VAC integration into a novel early goal-directed therapy would be to recouple the heart and circulation toward optimal efficiency with Ea/Ees in-between a “normal range” estimated in healthy humans ***, as reported in ref (3). N, normal; Ea, arterial elastance; Ees, end-systolic elastance; ESPVR, end-systolic pressure–volume relationship; EDPVR, end-diastolic pressure–volume relationship; PE, potential energy; SW, stroke work; Pes, end-systolic pressure; Ves, end-systolic volume.

A traditional hemodynamic perspective of acute sepsis characterizes this syndrome as a pattern of blood volume redistribution resulting in low blood pressure associated with variable fluid and vasopressor responsiveness (10, 11). However, on a case-by-case basis, at least 10% of patients do have subnormal circulatory parameters at admission, and up to 40% exhibit sepsis-associated cardiac dysfunction (12–14) (Figure 2).

VA uncoupling is frequently observed in the early phase of septic shock. In the literature, the prevalence of uncoupling (e.g., defined by Ea/Ees ratio >1.36) in critically ill patients varies from 25% to 85% (16–19) (Figure 2) and is associated with higher mortality (18). Also, a trend of distortion in the Ea/Ees ratio, outcome-related, has been recently observed in a small cohort study (20). Lowering the Ea/Ees ratio between 0.6 and 1.2—a range generally observed in healthy humans—could be a target goal (Figure 2) (3). VA uncoupling can exist despite normal or subnormal blood pressure (16). Hence, sizing shock resuscitation after volume optimization solely to a 65 mmHg MAP level for all patients and regardless of any cardiac function assessment does not seem to be adequate. This could lead to premature use and higher doses of vasopressors and an inappropriate increase in Ea (and afterload) as well as an increase in stroke work, globally reinforcing VA uncoupling. Also, it should be noted that VA uncoupling can be observed in patients with “normal” LVEF, enhancing the necessity of a more accurate circulatory assessment.

## Hemodynamic drug impacts on VAC specifically relevant to sepsis

Few drugs are currently available for the circulatory support of septic shock, and one does not fit all. To stretch a point, inotropes enhance Ees, whereas vasopressors specifically increase Ea, but several drugs share both abilities. Some patients do sometimes require more inotropism than vasopressors, because around 40% exhibit sepsis-induced myocardial depression (21). Many of the interventions frequently performed in these patients can directly or indirectly affect the arterial load and ventricular function, making the use of standardized strategies in sepsis difficult (20, 21). Thus, the assessment of VAC is of interest to critically ill patients in selecting correctly the appropriate drug for personalized sepsis resuscitation. In this section, we will discuss the existing literature on VAC and representative circulatory agents that are used or are under evaluation to be used in septic shock.

### Norepinephrine

Norepinephrine (NE) is a natural catecholamine that exerts a dose-dependent vasoconstrictive effect by its agonism on α-adrenergic receptors present throughout the vasculature (27). NE has also an inotropic effect, even if to a lesser extent, on β-adrenergic receptors (24). NE targets not only the arterial tree but also the venous blood reservoir, leading to the recruitment of an initially unconstrained blood volume toward a so-called constrained volume (27, 28). Moreover, NE also rises blood pressure of the Valsalva sinus and induces β_2_-mediated vasodilation of coronary arteries, overall normalizing coronary blood flow and contributing to an improved cardiac function (29). Finally, NE exerts a direct inotropic effect on the myocardium through both α_1_ and β_1_ stimulation (30). Thus, circulatory effects of NE in sepsis can contribute to the three main areas of hemodynamic homeostasis: i.e., pre- and afterload and cardiac inotropism. Unfortunately, the beneficial effects and responsiveness of NE also depend on the course of sepsis, timing of administration, actual vascular resistance, and the prior cardiac status of the patient. Specifically, a preexisting heart failure, where any increase in afterload may impair ventricular ejection, is potentially problematic for NE use. This suggests the exploration of VAC as a reliable parameter to explore the global circulatory effects of NE in sepsis, even though very few studies have explored this issue with somewhat contradictory observations. Zhou et al. (31) showed that, in 34 septic shock patients, NE infusion tends to normalize VAC by elevating Ees to a higher magnitude than Ea. Moreover, patients with Ea/Ees ratio close to 1.0 are more sensitive to increase their SV with NE infusion. In contrast, Guarracino et al. observed that, in a prospective cohort study, NE infusion of septic shock patients enhanced Ea with minimal effects on Ees, leading to a worsening of VA uncoupling and loss of left ventricular efficiency (25). In this study, nearly half of the patients did not respond to NE, whereas responding patients presented with less elevated Ea/Ees values. Thus, quite often, rather than a consequence of NE infusion, VA uncoupling was a cause of NE inefficiency. This evidence-based finding argues for guiding appropriate circulatory treatment during shock states by monitoring VAC because it may be an indirect marker of myocardial stunning in sepsis. This is reinforced by the validation of the dynamic arterial elastance

$$\left(\mathrm{Eadyn}=\frac{\mathrm{pulse}\;\mathrm{pressure}\;\mathrm{variation}\;(\mathrm{PVV})}{\mathrm{stroke}\;\mathrm{volume}\;\mathrm{variation}\;(\mathrm{SVV})}\right)$$

 an indirect marker of VAC, as a predictor of successful NE weaning (22). The higher the Eadyn and left ventricular efficiency, the lower the VAC (23).

### Dobutamine

Dobutamine is an old synthetic catecholamine exerting powerful inotropic effects through its agonism on β_1_ receptors and is still widely used in circulatory shock (25). The surviving sepsis campaign (SSC) suggests adding dobutamine in cases of shock refractory to NE (1). Dobutamine used in patients with reduced CO and low LVEF in septic shock can contribute to restoring circulation. In this context, when MAP is sustainably low despite NE infusion, the addition of dobutamine resulted in a significant improvement of VAC (23). The latter came from a 35% reduction of the Ea/Ees ratio, mainly because of an increase in Ees with minimal effects on Ea, and a subsequent increase of SV (25). This “recoupling” of the ventricle and aorta may explain the suggested reduction of mortality in septic shock patients treated with dobutamine, as observed in a network meta-analysis (32). From a pathophysiological point of view, dobutamine improves VAC by both improving myocardial inotropism and by reducing afterload through a β_2_-mediated vasodilator effect, most notably in NE pre-treated patients in whom arterial resistance is often normal or supranormal. Nevertheless, starting dobutamine infusion in septic shock must be preceded by an echocardiography assessment and preferably after VAC exploration, to avoid potentially harmful administration in patients without cardiac dysfunction. This point may explain the inefficiency or even increased mortality rates with dobutamine use in sepsis, as reported by several meta-analyses and retrospective studies (33, 34). Finally, it should be noted that, because of its positive chronotropic effects, dobutamine could lead to ventriculo-arterial uncoupling. Indeed, excessive, and inappropriate tachycardia could lead to an increase in arterial elastance by increasing the telesystolic pressure (early dicrotic wave).

### Levosimendan

Levosimendan is a “new” generation inodilator with different pharmacodynamic properties than catecholamines, since it does not interact with adrenergic receptors (35). Its mechanisms of action involve sensitization of calcium–troponin interactions and activation of potassium channels, both of which allow an increase in inotropism and vasodilation (25). Benefits have been demonstrated with levosimendan infusion in patients with chronic congestive heart failure and may also be beneficial in perioperative cardiac surgery, although these results need to be confirmed (36–40). However, the use of levosimendan in septic shock is highly controversial, as testified by the results obtained by Gordon et al. (41) in a randomized trial of 516 septic shock patients where no benefit was observed and higher rates of supraventricular arrhythmia and issues in weaning from mechanical ventilation were revealed. Nevertheless, in this trial, the studied population was not selected for sepsis myocardial failure, and less than 20% of patients were pretreated with dobutamine in addition to NE, potentially explaining both the lack of a positive impact and the most frequent side effects of levosimendan. Recent meta-analyses are more supportive of the use of levosimendan in septic shock with higher CO and lactate blood level reduction in comparison with dobutamine (26) but without an obvious drop in mortality rates (42). In an animal model of septic shock induced by LPS administration, levosimendan, in contrast to cAMP-dependent inotropes (dobutamine and milrinone), partially restored left ventricular contractility by increasing Ees and improved myocardial relaxation and cardiac filling, without altering vascular properties (Ea not altered by levosimendan administration) (43). Although efficient in rebalancing the Ea/Ees ratio of patients with human ischemic cardiomyopathy, the effect of levosimendan on VAC was unfortunately never directly explored in human septic shock (44). Despite similar effects on VAC as dobutamine can be expected with levosimendan, further studies are warranted to explore this topic.

### β-blockers

Unlike the above drugs, β-blockers (BBs) inhibit β1 receptors but may also be of interest during septic shock under specific conditions of circulatory failure. Two recently developed BBs stand out from the others because of their pharmacokinetics and pharmacodynamics, i.e., esmolol and landiolol (45). These two drugs do have powerful selective β1-blocking effects with minimal β2-blocking impact and a short elimination half-life, even more pronounced for landiolol. In septic shock, esmolol and landiolol have been demonstrated to reduce heart rate, without impacting MAP in a majority of patients (46, 47). This does not oppose the positive effects of catecholamines and inodilators discussed above but further supports the need for VAC assessment of patient selection. Different profiles of septic patients can explain whether tachycardia is adaptive or maladaptive and whether blocking or stimulating the adrenergic system is required. In a cohort of 45 patients, Morelli et al. (48) observed that a reduction of heart rate using esmolol resulted in a decrease of Ea with a concomitant increase of SV. Indeed, tachycardia induces an acceleration of the pulse wave reflection (dicrotic wave), generating an additional pressure in telesystole (and no longer in protodiastole). Thus, in the case of maladaptive tachycardia, the end-systolic pressure is increased, as is Ea (=ESP/SV). These patients presented a preserved LVEF above 50%, suggesting that the beneficial effects address patients without sepsis-induced myocardial depression. Although these results are of great interest, particularly because of the clinical improvement with reduced NE requirements, they also need to be confirmed by further analyses as to the direct effects on VAC (49). Indeed, the authors focused on Ea without correctly evaluating Ees, whereas an altered myocardial function by β-receptor blockade cannot be ruled out (50). In a *post hoc* analysis and to overcome this limitation, these authors suggested the use of the systolic–dicrotic notch pressure difference as an indirect tool for evaluating VAC and representative of the efficiency of myocardial contraction (systolic pressure) in opposition to a given afterload (dicrotic notch pressure). In this context, low values of systolic–dicrotic notch pressure difference were related to VA uncoupling and could predict at baseline the ability of β-blockers to improve cardiac efficiency with a heart rate reduction, discriminating adaptive from maladaptive tachycardia (48). Although attractive and simple, such an approach needs to be confirmed, notably because of the number of limitations regarding the reliability of arterial waveforms in distal locations of the heart and with some settings (51). In summary, BBs may be of interest in septic shock patients when a maladaptive tachycardia impedes left ventricular filling and limits ventricular ejection. In that event, lowering heart rate recouples the ventricle with the arterial tree by maximizing ventricle performance and with little effect on arterial compliance (notably when using β_1_ selective antagonists, such as esmolol and landiolol). A still unresolved genuine challenge is the fine-tuning selection of candidate patients.

### Other drugs and molecules

Arginine vasopressin (AVP) and apelins (APL) belong to a family of neuropeptides with pleiotropic, often counter-regulatory, cardiovascular or related effects (52). AVP has a somewhat recognized added value with specific catecholamine-sparing vasoconstrictive effects in septic shock (1, 53), without a direct effect on the heart identified. From a physiological point of view, AVP has a vagal- and/or sympathetic-independent cardiodepressant effect (54). More specifically, AVP drops the left ventricular ESPVR slope, shifting it to the right (55). Pathophysiologically, AVP-specific V1 receptor (V1aR) expression is increased in failing hearts, but this is related to an impaired cardiac function independently of any effect on the vascular system (56). AVP-specific V2 receptors (V2aR) in the heart can have a positive inotropic effect but needs gene transfer overexpression (57). APL are still molecules in development, but in the preclinical stage, they improve cardiac function, diuresis, and the cardiorenal axis, counteracting the AVP activities (58–60). However, the inotrope ability of APL is still a matter of debate (despite several demonstrations *in vivo* and in isolated hearts *ex vivo*). The essential absolute gain of CO in response to APL-13 isoform is clearly linked to SV improvement without significant chronotropic effect but with an attributable contribution of the underlying degree of total peripheral resistance. VAC, as well as ventricular mechanical efficiency, are improved by more than 40% with APL-13 infusion in experimental ischemic failing hearts with an induced increase of the Ea/Ees ratio (61). APL-13 also clearly improves the left ventricular P/V relationship and reduces Ea in small and large animal models of septic shock (58–60). Essentially studied at the preclinical level, APL have been also assayed in humans (both healthy volunteers and patients with pulmonary arterial hypertension and heart failure). In recent years, several pharmaceutical companies have worked on APL receptor agonists such as PEGylated, N-linked hydroxypyridine-based APL, and AMG-986 agonists. The latter was a first-in-man trial using an orally delivered molecule that was recently released, showing a potentially interesting rise of SV in chronic heart failure patients (62–64).

Angiotensin II has been recently added as a recommended third-line rescue vasopressor for refractory septic shock in the SSC guidelines (1). This is a powerful vasoconstrictive agent, devoid of any direct cardiac effect, increasing ventricular stroke work and load. As to VAC, angiotensin II is an improbable candidate to restore Ea/Ees balance in septic patients with an uncoupling profile as it is more likely to increase Ea. This additionally argues for selecting patients on the potential of benefiting or not from this drug.

## Conclusion

In conclusion, VAC is a valuable tool for understanding the interaction between the myocardial contractile function and the load opposed by the arterial circulation. Although many data are encouraging, the benefit of such an approach to in-hospital outcomes should be confirmed in the future. Additional proof of applicability and optimal targets are needed to consider VAC at the bedside in complex hemodynamic conditions, such as septic shock, where tachycardia and pharmacologic increase in vascular resistance contributes to ventriculo-arterial uncoupling in associated but overlooked myocardial injury. This would support the decision-making strategies of intensivists in today’s world of individualized medicine.
